# Optimization of tamoxifen-induced Cre activity and its effect on immune cell populations

**DOI:** 10.1038/s41598-020-72179-0

**Published:** 2020-09-17

**Authors:** Rachel S. Donocoff, Nato Teteloshvili, Hyunsoo Chung, Rivka Shoulson, Remi J. Creusot

**Affiliations:** 1 Institute of Comparative Medicine, Columbia University Medical Center, Columbia University, New York, NY 10032 USA; 2 Department of Medicine, Columbia Center for Translational Immunology and Naomi Berrie Diabetes Center, Columbia University, New York, NY 10032 USA; 3 Columbia Center for Translational Immunology, Columbia University Medical Center, 650 W. 168th Street, New York, NY 10032 USA

**Keywords:** Biological techniques, Immunology

## Abstract

Tamoxifen (TAM) inducible Cre recombinase system is an essential tool to study gene function when early ablation or overexpression can cause developmental defects or embryonic lethality. However, there remains a lack of consensus on the optimal route and dosage of TAM administration in vivo. Here, we assessed dosage and delivery of TAM for activation of Cre in immune cell subsets assessed longitudinally and spatially using transgenic mice with ubiquitously expressed Cre/ER and the Cre-inducible fluorescent reporter YFP. After comparing two TAM delivery methods (intraperitoneal versus oral gavage) and different doses, we found that 3 mg of TAM administered orally for five consecutive days provides maximal reporter induction with minimal adverse effects in vivo. Serum levels of TAM peaked 1 week after initiating treatment then slowly decreased, regardless of dosing and delivery methods. TAM concentration in specific tissues (liver, spleen, lymph nodes, and thymus) was also dependent on delivery method and dose. Cre induction was highest in myeloid cells and B cells and substantially lower in T cells, and double-positive thymocytes had a notably higher response to TAM. In addition to establishing optimal dose and administration of TAM, our study reveals a disparate activity of Cre in different cell immune populations when using Cre/ER models.

## Introduction

Tamoxifen (TAM)-inducible Cre/loxP is one of the most widely used inducible systems for gene regulation. This is in part due to the fact that it enables gene control both spatially and temporally. The inducible Cre/loxP estrogen receptor (ER) transgenic system is used to study both ubiquitous and tissue-specific gene function through the expression of Cre recombinase fused to the G525R mutant form of the mouse estrogen receptor 1 (Esr1) to create a Cre/ER fusion protein^1,2^. At physiologic concentrations, the mutated ER does not bind its natural ligand (17β-estradiol), but it is activated when bound to the functional TAM metabolite 4-hydroxytamoxifen (4-OHT)^1–3^*.* Due to prohibitive costs of using 4-OHT, TAM is a practical synthetic substitute to induce gene recombination^4^, since it is converted into 4-OHT in the liver^5,6^. Mature mice metabolize TAM to 4-OHT rapidly, producing comparable serum levels of the two compounds^7,8^. Cre/ER recombinases remain unexpressed until activated by the TAM metabolite, which provides external and time-specific control of Cre activity. This system is used to address the function of specific genes^3,9–11^, including those relating to immune cells. This system is often required to circumvent issues of embryonic lethality when ablation of the gene of interest affects fetal development in vivo.

In order to determine the pattern of Cre activity and quantify the percentage of Cre-expressing cells, various mouse lines have been engineered with a transgenic reporter. The reporter is not expressed under physiologic conditions, but TAM-induced excision of the loxP-flanked STOP cassette upstream enables its transcription^12^. Reporter lines that are most widely utilized contain a fluorescent protein (e.g. YFP) in the ROSA26 locus to verify efficient expression in all cell types, including their developmental stages^13,14^. These lines are mainly used for lineage tracing, taking advantage of permanent induction of the reporter even after TAM delivery is discontinued. The Cre/ER transgene is expressed under either tissue-specific or ubiquitous promoters, such as CAG, which is a composite of CMV enhancer element, chicken β actin promoter and the splice acceptor of the rabbit β globin gene^15,16^. In such a ubiquitous system, whether all cells express Cre/ER to the same level and induce loxP recombination to the same extent remains unclear. Induction and consequent expression may depend on many factors, including the nature of the gene in question or host cell type, and requires further investigation^15^. Several studies have addressed conditional gene deletion and overexpression within the adaptive and innate immune system in order to analyze the effect on phenotypic changes in immune cells^17,18^. Distinct populations of immune cells are targeted depending on the tissue-specific Cre-transgenic mouse. Selection of which promoters to use is based on Cre expression in specific cells and tissues^19^.

Analyzing the efficacy of induction for published Cre lines is difficult, in part due to the wide range of application protocols that include diverse dosages and preparations of TAM, and various routes of administration. To gain information about TAM usage at an institutional level, a survey was conducted amongst investigators utilizing tamoxifen for gene induction studies at Columbia University, and the response indicated that the formulation, route of administration and dosage were varied (Suppl. Information Fig. S1). The results of the survey and current literature reveal that there is no consensus on the best conditions for TAM delivery. It is also unclear whether TAM- affects different cell types uniformly to induced Cre activity in a ubiquitous system. The majority of studies utilized intraperitoneal (IP) injections of TAM, while side-by-side comparisons with other routes of delivery such as oral (per os, PO) have not been thoroughly investigated^4,20,21^. In past studies, TAM dosage has been altered based on animal age, target tissue, and other factors^21^. In general, it has been noted that older transgenic mice require higher TAM doses and longer treatment periods, depending on the mode of administration, to achieve satisfactory induction of Cre/ER activity^21^. Several studies have used TAM to decipher the functional relevance of conditional gene deletion in specific immune cell subsets through temporal control of Cre expression. However, there remains limited knowledge on optimal dosage and delivery of TAM for efficient Cre induction in hematopoietic cell lineages^22,23^.

In this study, we aimed to determine which combination of dosage and route of TAM administration achieves the most effective induction of Cre based on YFP reporter expression. Additionally, we investigated the effect of TAM on the clinical picture of the mice including clinical pathology and histopathological changes. TAM concentration in various organs and YFP induction rate in different immune cell populations were also measured.

## Results

### TAM-mediated induction of Cre within CD45+ cells is dependent on dosage and route of administration

**Figure 1:**
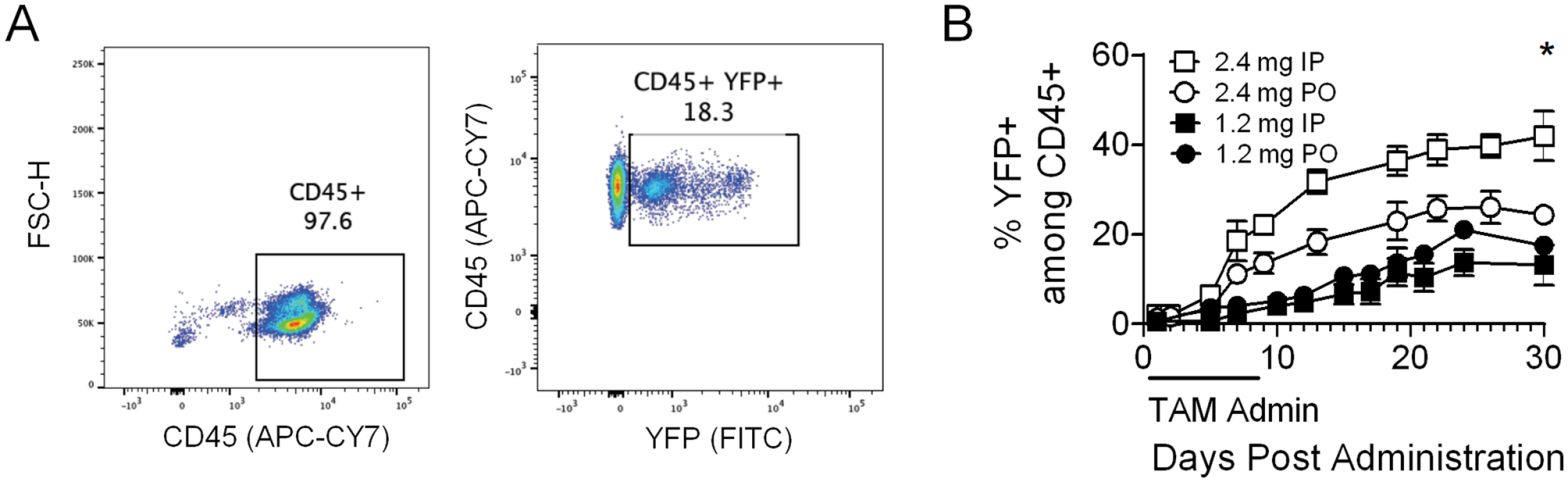
TAM-mediated induction of YFP reporter. (**A**) Representative scatter plots of the gating strategy to identify Cre activity by the expression of the YFP reporter among CD45+ cells on day 5 after initiation of treatment with 3 mg TAM PO for 5 days. (**B**) Testing of TAM dosage (2.4 mg and 1.2 mg) given IP and PO every other day for 5 days (n = 3–5/group). Data are presented as mean with standard error of the mean (SEM). Mixed model ANOVA test with repetitive measures was carried out to identify significant differences when testing different concentrations and delivery methods. *p ≤ 0.05.

The ubiquitous inducible CAG-Cre/Esr1 system in conjunction with the Cre-inducible YFP reporter provides a useful model to perform longitudinal analyses using whole blood to optimize TAM dosage and route of administration. The Cre/Esr1 is constitutively expressed, but only translocates to the nucleus when bound to TAM’s metabolite 4-OHT where it then recombines LoxP sites. In the case of this YFP reporter, the Cre-mediated recombination removes a STOP codon upstream of the YFP gene, allowing its expression under the Rosa26 promoter. We treated adult mice with 2.4 mg and 1.2 mg of TAM via IP or PO administration every other day for five days and measured the induction of Cre/YFP by TAM on hematopoietic cell lineages (CD45+ cells) in peripheral blood cells (PBC) (Fig. 1A). YFP induction was analyzed for 30 days via collection of peripheral blood samples at least twice a week for flow cytometry analysis. We noted a significant increase in the frequency of YFP positive CD45+ cells when treating mice with 2.4 mg TAM, reaching 40% by day 30 under the best conditions (Fig. 1B). Overall, induction of YFP was higher when giving mice 2.4 mg TAM compared to 1.2 mg (either IP or PO). These data indicate that induction of Cre activity by TAM is dose-dependent and is enhanced when delivered IP.

### Dosage and route of TAM administration correlates with serum concentration and morbidity in mice, especially when administered IP

**Figure 2:**
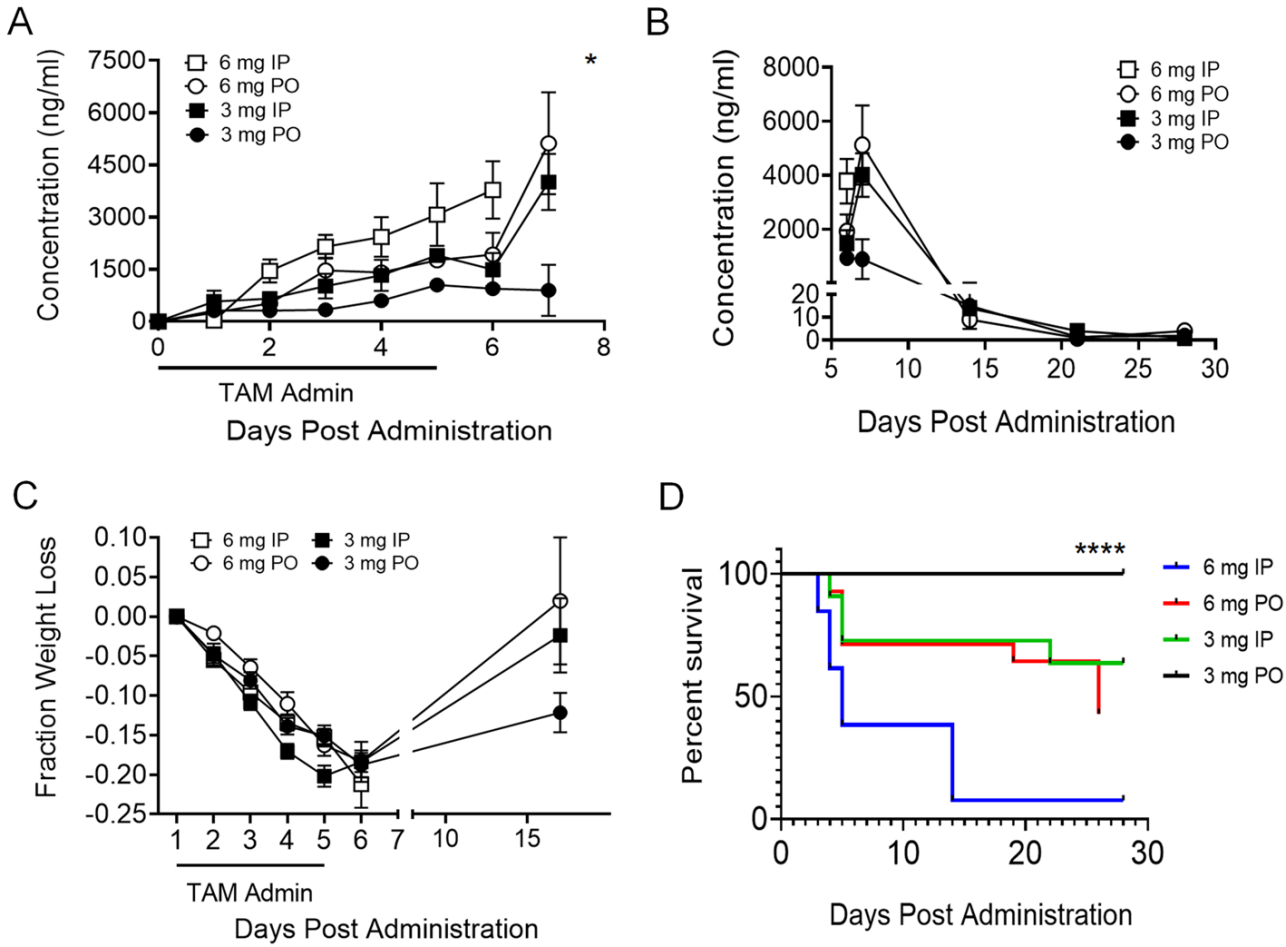
Dosage and route of TAM correlate with serum concentration and morbidity in mice. (**A**) TAM concentration measured in serum daily over a period of 7 days following IP or PO treatment of mice daily for 5 days (mean ± SEM of 2 pools of 5 mice). (**B**) TAM concentration measured in serum over a period of 28 days following IP or PO treatment of mice (mean ± SEM of 2 pools of 2 mice). (**C**) Fraction of weight lost in mice following TAM treatment for 5 consecutive days, either IP or PO (n = 10/group). Mice that received treatment via IP injection lost more weight and not all survived. Long-term weight could not be measured for the group of mice that received 6 mg TAM IP due to mortality. Mixed model ANOVA test with repetitive measures was carried out to identify significant differences when testing different concentrations and delivery methods. (**D**) Survival curve of mice following TAM treatment PO or IP with different concentrations. Data are presented as mean ± SEM. Black line shows percent survival of mice treatment with 3 mg PO, green line—3 mg IP, red line—6 mg PO and blue line—6 mg IP. Log rank test was performed on survival curve. *p ≤ 0.05, ****p ≤ 0.0001.

In an effort to achieve greater levels of Cre activity, we explored higher TAM doses. We analyzed TAM concentration in serum samples to determine the relationship between TAM-mediated induction of Cre and its accumulation and clearance from serum. TAM concentration was measured daily over a period of 7 days and then weekly for a total of four weeks following a 5-day treatment with 3 mg or 6 mg TAM daily IP or PO. TAM serum concentration was highest on day 7, with significant accumulation when treating mice with 6 mg either IP or PO (Fig. 2A). TAM clearance was monitored over a period of 28 days and was eliminated by day 25 (Fig. 2B). The observed maximum induction of the YFP reporter from the 6 mg dose of TAM administered IP coincided with the highest concentration of TAM measured in serum. Animals treated with TAM, regardless of the dose and route of delivery, experienced marked weight loss following 5 days of treatment, which correlated with TAM serum concentrations following treatment (Suppl. Information Fig. S2). Animals that received the lower dose (3 mg) either IP or PO or the higher dose (6 mg) of TAM PO partially regained weight lost (Fig. 2C). Notably, many of the mice that lost the greatest percentage of weight were in the group that received high dose (6 mg) TAM IP, but this is not reflected in the data because the majority of mice in this group had to be euthanized due to morbidity and excessive weight loss (Fig. 2D). Increased mortality was noted with high dose TAM and IP administration, most dramatically when the two were combined, and therefore we were unable to document long-term weight loss or recovery in this group. This observation indicates an adverse effect of TAM given at a high dose IP.

### High dose of TAM induces high Cre activity, but is also accompanied by hepatic changes

**Figure 3:**
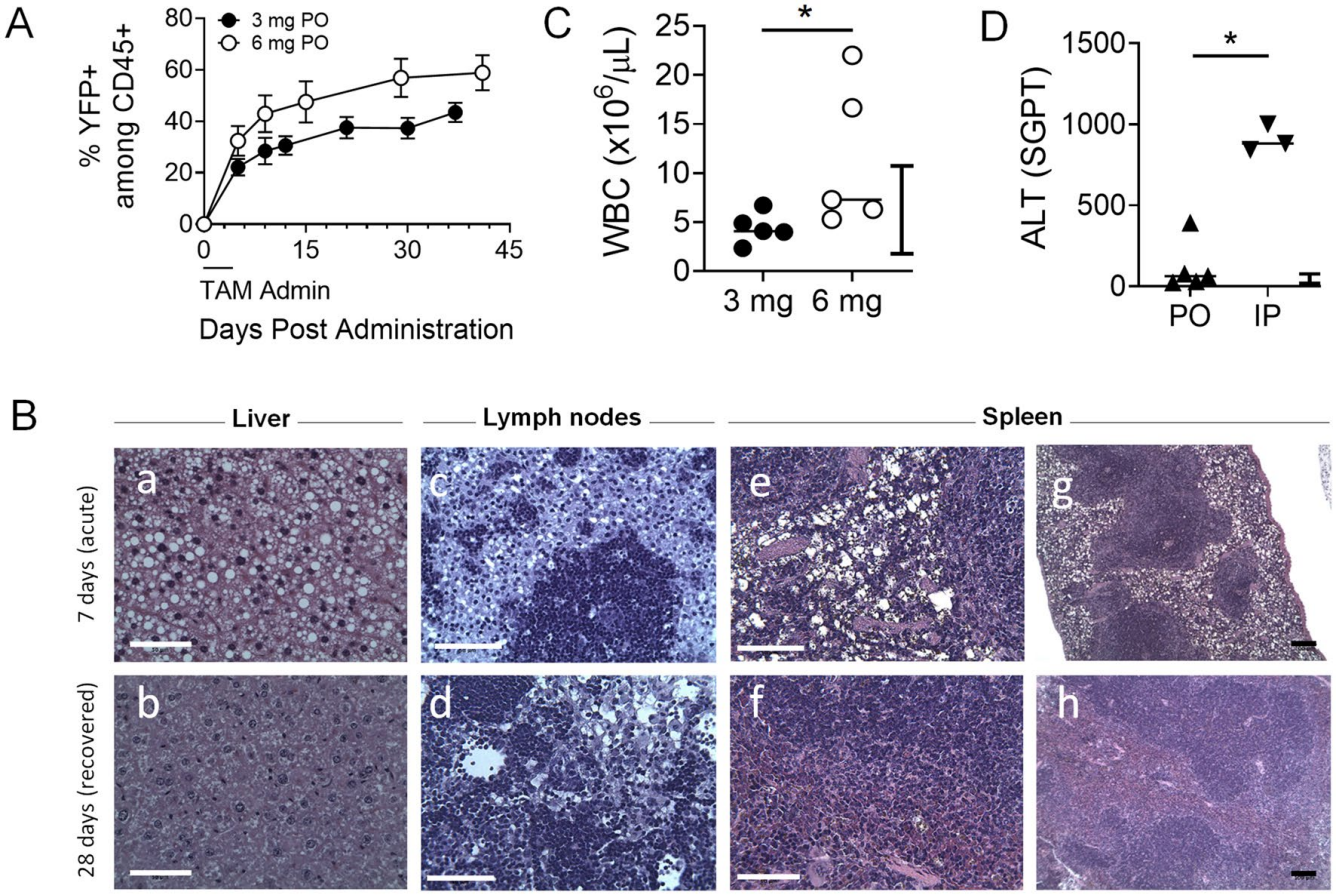
Treating mice with high dose of TAM increases YFP induction among CD45+ cells but also results in liver injury. (**A**) Frequency of YFP-expressing CD45+ cells after PO administration of TAM at 3 or 6 mg. (**B**) Histopathology analysis on fixed liver (a,b), lymph nodes (c,d) and spleen (e–h). The upper row reveals apparent macrophage lipidosis during acute phase (on day 7 after 5 IP treatments with 3 mg TAM). The lower row shows no macrophage lipidosis present in any tissues on day 28 in mice that fully recovered. White scale bars (a–f) indicate 50 μm and black scale bars (g,h) indicate 100 μm. (**C**) White blood cell (WBC) count and (**D**) Alanine aminotransferase (ALT) test measuring serum glutamic-pyruvic transaminase (SGPT) from mice treated with 3 mg or 6 mg TAM PO and IP. Data are presented as mean ± SEM (n = 3–5 mice or pools of mice/group). Bars indicate normal reference range for WBC and ALT in mice, provided by manufacturer and based on calibration. Man-Whitney test for used for unpaired sample comparisons. *p ≤ 0.05.

Once we determined that administering TAM PO was safer than the IP route, we compared two doses (3 mg and 6 mg) given PO to further assess the effect of TAM on mice. In addition, we assessed alanine amino transferase (ALT) levels from PO and IP mice to confirm adverse effect of TAM when delivered IP on liver samples. The two doses had different effects on morbidity and mortality. TAM induced ~ 55% YFP+ cells when given 6 mg PO, more than 3 mg PO, and induction of the reporter plateaued 30 days post treatment regardless of dose (Fig. 3A). Animals were euthanized and necropsied either 7 or 28 days post initiation of treatment. Lymph nodes (LNs), spleen and liver were fixed in formalin and histopathologic analysis was performed. We found cytoplasmic vacuolation, identified on H&E staining, in all these tissues, consistent with intracytoplasmic lipid accumulation (Fig. 3B). These findings led to a presumptive diagnosis of hepatic lipidosis in the livers of animals treated with all doses of TAM necropsied 7 days after initiation of treatment^24^. Vacuolation was also noted in the macrophages of the splenic and LN sinusoids in mice necropsied 7 days following the start of TAM treatment (Fig. 3B(c–f)), which is consistent with the hepatic lipidosis^24,25^. However, vacuoles were not detected in these organs 28 days post treatment (Fig. 3B), which suggests that animals were able to metabolize and clear the lipid accumulation, likely originating from the TAM delivery vehicle (corn oil), at least 4 weeks post treatment. Thus, these pathologies appeared transient and normalized unless the mice succumbed from acute toxicity. To determine the degree of liver damage and the effect on inflammation, we measured levels of ALT and white blood cell (WBC) count. Leukocytosis (presence of circulating leukocytes above normal range) was found in PO treated animals but only with 6 mg (Fig. 3C), while all IP treated animals had leukocytosis regardless of dose (range 9.5–82, not shown). Higher levels of ALT were detected in animals treated with TAM IP, with low and high doses combined (Fig. 3D). These results identify the toxic effects of higher doses of TAM administered, particularly when given IP.

### Reaching an optimal TAM dose and delivery route results in efficient induction of Cre activity with minimal adverse effects

**Figure 4:**
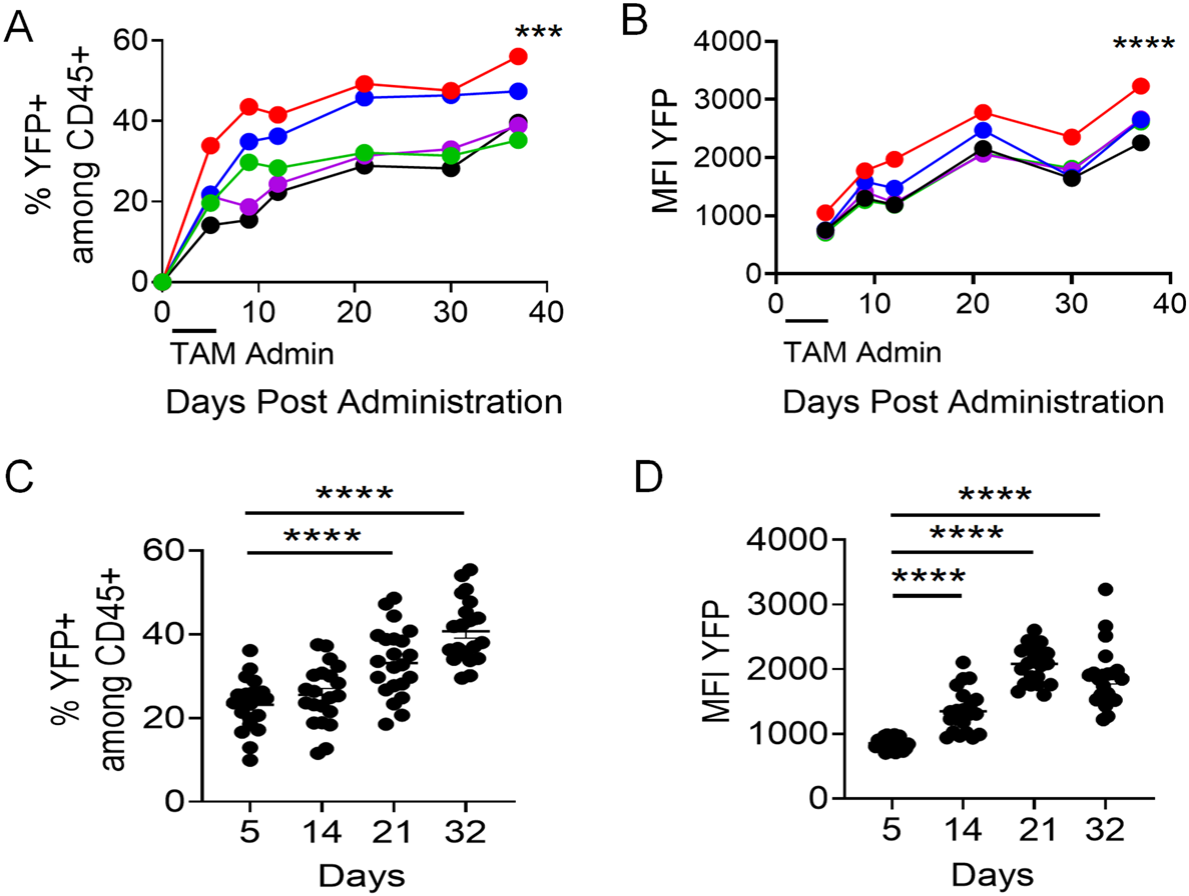
Optimized dosage and route of TAM administration induces gradual increase in YFP expression over time. (**A**) Frequency and (**B**) MFI of YFP expression within peripheral blood CD45+ cells analyzed at different time points (n = 5). Different colors represent individual mice. Validation with another larger cohort of mice (n = 21) showing (**C**) frequency (Day 5—23.2 ± 6.1%, Day 14—25.2 ± 7.2%, Day 21—32.9 ± 8%, Day 32—40.7 ± 7.6%) and (**D**) MFI of YFP expression (Day 5—855.9 ± 89.7, Day 14—1,354.5 ± 337.3, Day 21—2,104.7 ± 275.6, Day 32—1875.9 ± 474.3) within peripheral blood CD45 + cells at different time points after the beginning of treatment. YFP MFI was measured on YFP+ cells. In both cohorts, mice were treated for 5 consecutive days with 3 mg of TAM administered PO. Mixed model ANOVA test with repetitive measures was performed to identify significant differences following 3 mg TAM treatment PO (**A**,**B**). Data are presented as mean ± SEM. Non-Paired T test for used for unpaired sample comparisons (**C**,**D**). ***p ≤ 0.001, ****p ≤ 0.0001.

Among the conditions tested for efficient Cre induction and YFP expression in mouse hematopoietic cells, 3 mg TAM delivered to animals PO for 5 consecutive days was determined to be optimal. We measured the mean fluorescent intensity (MFI) and frequencies of YFP-expressing CD45+ cells by flow cytometry in PBC of animals twice weekly beginning 5 days following daily TAM treatment. As expected, we observed induction of YFP as early as day 5 of treatment in both MFI levels and percentage of YFP + cells, with consistent trends in individual mice within the same experimental group (Fig. 4A,B). There was no mortality or substantial morbidity noted in these mice, and frequencies of YFP+ cells in another cohort of mice reached significance at day 21 and day 32, with maximal induction of ~ 40% (Fig. 4C) and MFI levels of YFP were significantly increased starting 14 days post treatment in grouped mice (Fig. 4C,D). Cre activity, as measured by expression of the YFP reporter, was effectively induced using 3 mg of TAM PO without the morbidity and mortality observed on experimental animals at higher dosages and IP administration. Moreover, the effect of TAM was long lasting, and Cre induction continued to increase 3–4 weeks after discontinuing TAM administration. This suggested to us that TAM may accumulate in tissues for long-term availability, and excessive accumulation could have serious adverse effects.

### High concentration of TAM administered IP, but not PO, accumulated in the liver and spleen of mice

**Figure 5:**
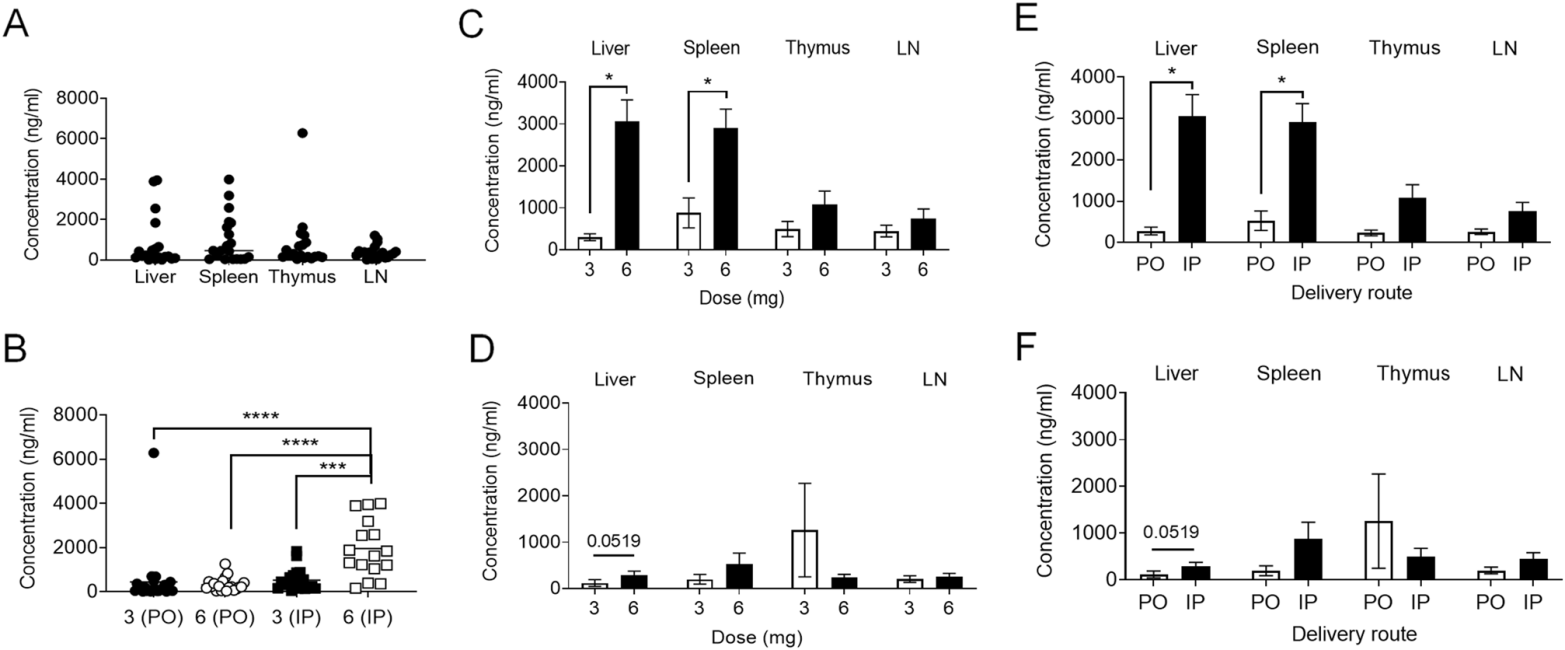
Concentration of TAM measured in tissues. IP injection of TAM results in greater TAM concentration in tissues than PO administration. Comparison of TAM concentration between different tissues regardless the dose and delivery route of TAM (**A**; n = 21/group), and when given IP versus PO 3 mg and 6 mg dose from pooled tissues (**B**; n = 21/group). Comparison of TAM concentration given by IP (**C**) and PO (**D**) route (n = 4–5/group). Comparison of tissue TAM concentration between IP injection and PO when give TAM with high dose—6 mg (**E**) and low dose—3 mg (**F**) (n = 4–5/group). Data are presented as mean ± SEM. Mann–Whitney test was used for unpaired sample comparisons. *p ≤ 0.05, ***p ≤ 0.001, ****p ≤ 0.0001.

To ascertain whether TAM dosing and delivery route affects its accumulation in different tissues, we treated mice with 3 mg and 6 mg of TAM for 5 days either PO or IP. We euthanized mice 7 days and 28 days (3 mg IP and 6 mg PO treated mice) after initiating treatment, isolated liver, spleen, thymus and LNs to analyze the concentration of TAM in these tissues. Within the tissue samples, concentration of TAM measured on day 7 post-treatment did not differ among liver, spleen, thymus and LN tissues when not differentiated by dose or administration route (Fig. 5A), however the highest concentration of TAM was detected in animals dosed with 6 mg IP regardless of the source of the tissue (Fig. 5B). Accumulation of TAM in liver and splenic tissues significantly increased with the 6 mg dose compared to 3 mg IP injection (Fig. 5C), but not with PO delivery (Fig. 5D). Additionally, IP delivery of the drug at 6 mg resulted in increased tissue TAM concentration compared to PO (Fig. 5E), but this was not evident with the 3 mg dose (Fig. 5F). Thus, high dose of TAM injection results in accumulation in tissues when administered IP but not PO. TAM was cleared from all tissues measured 28 days after initiation of TAM administration, with all concentrations measuring less than 0.2 ng/mL (data no shown).

### TAM differentially affects Cre activity in immune populations

**Figure 6:**
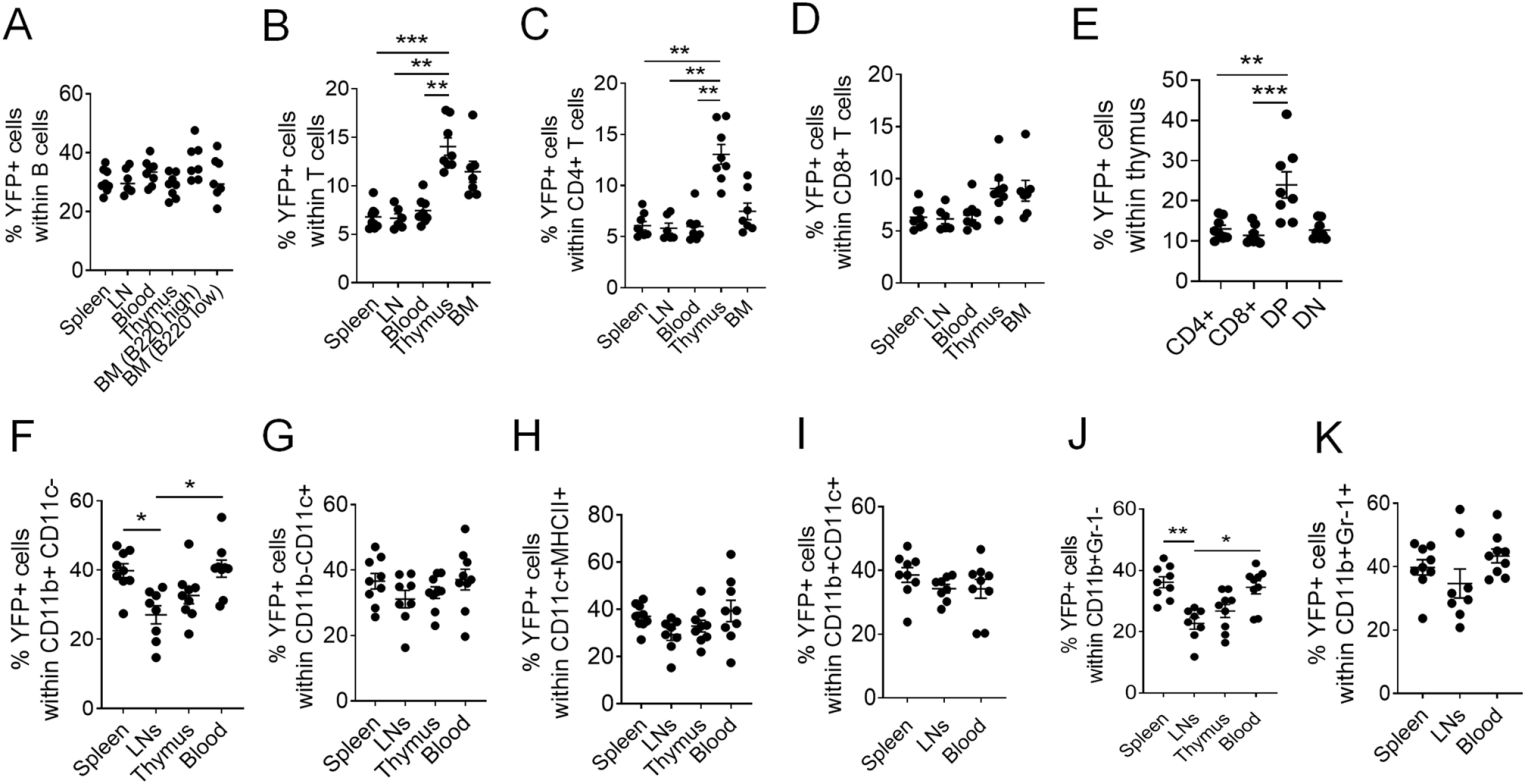
Highest induction of Cre in myeloid cells, and among T cells, in DP thymocytes. Frequency of YFP expressing cells (%) in (**A**) B cells (B220+ CD3−), (**B**) T cells (B220− CD3+), (**C**) CD4+ T cells (B220− CD3+ CD4+ CD8−), (**D**) CD8+ T cells (B220− CD3+ CD4− CD8+), (**E**) single positive CD4+ /CD8+, double positive (DP) and double negative (DN) thymocytes, (**F**) CD11b+ CD11c− macrophages, (**G**) CD11b− CD11c+, (**H**) CD11c+ MHCII+ and (**I**) CD11c+ CD11b+ dendritic cell populations, (**J**) CD11b+ Gr-1− and (**K**) CD11b+ Gr-1+ cells. Data was collected from 8 mice (CAG-Cre+) treated with TAM 3 mg PO. See Suppl. Information Fig. S2 for cell gating strategy, including BM B220 high and low cell gating. Data are presented as mean ± SEM. Mann–Whitney test was used for unpaired sample comparisons. *p ≤ 0.05, **p ≤ 0.01, ***p ≤ 0.001.

CAG promoter drives ubiquitous gene expression in a variety of tissues, including hematopoietic CD45+ cells, which are comprised of lymphoid and myeloid lineages. To determine whether induction of Cre activity by our optimized condition (3 mg TAM dose PO) was comparable between lineages of immune populations, we measured YFP reporter induction across various lymphoid tissues, including spleen, LNs, thymus and bone marrow samples in addition to blood. YFP expression in B cells was comparable across different tissues (~ 30%) (Fig. 6A, Suppl. Information Fig. S3). YFP induction was considerably lower in T cells (~ 7%) in secondary lymphoid tissues, albeit higher in primary lymphoid tissues, particularly in the thymus (~ 14%) (Fig. 6B), which was attributed to CD4+ rather than CD8+ T cells (Fig. 6C,D) and to DP thymocytes more specifically (Fig. 6E). The highest level of YFP induction (~ 40%) was in myeloid cells, comprising many antigen-presenting cell (APC) populations, such as macrophages, monocytes, dendritic cells, and granulocytes. For some of these subsets, induction was significantly higher in spleen and peripheral blood relative to LNs and thymus (Fig. 6F,K).

This disparity of YFP induction between immune cell populations may be contributed by different factors, including differential activity of the CAG promoter between cell types, different levels of expression of the endogenous ERs competitively binding TAM, and/or endogenous levels of Cyp2d, a cytochrome P450 family member that converts TAM to its active metabolite. First, because the mice used were either homozygous or heterozygous for the YFP reporter transgene (YFP+/+ and YFP+/− , respectively), we needed to ensure that the activity of the CAG promoter driving YFP expression was not confounded by the number of transgene copies. We analyzed the level of YFP expression from YFP+/+ and YFP+/−  mice separately, in peripheral blood during longitudinal analysis, in total splenocytes, and in different spleen populations at endpoint (Suppl. Information Fig. S4). The level of YFP did not significantly differ based on the number of transgene copies, and greater induction of YFP+ cells did not translate into higher YFP levels.

Second, in an initial attempt to address if expression of endogenous ER and Cyp2d genes could partially explain the disparity in YFP induction, we interrogated the ImmGen and BioGPS gene expression databases for relative expression of estrogen receptors Esr1 and Esr2 and Cyp2d9, 10, 11 (mouse ortholog of CYP2D6) (Suppl. Information Table S1A,B). These expression data are based on C57BL/6 mice, the same strain used in our studies. Esr1 is the most highly expressed ER in immune cells, but its expression does not negatively correlate with YFP induction, arguing against a dominant effect of endogenous ER expression (Suppl. Information Table S1A). Furthermore, expression of Cyp2d in immune cells and lymphoid tissues is negligible relative to expression in liver (Suppl. Information Table S1B). As these publicly available data could not help predict Cre/YFP induction, systematic determination of relative Cre/YFP induction in different populations, as done in this study for different immune cells, may be required.

### Different levels of Cre/YFP inducibility between immune cell populations are replicated by direct application of 4-OHT in vitro

**Figure 7:**
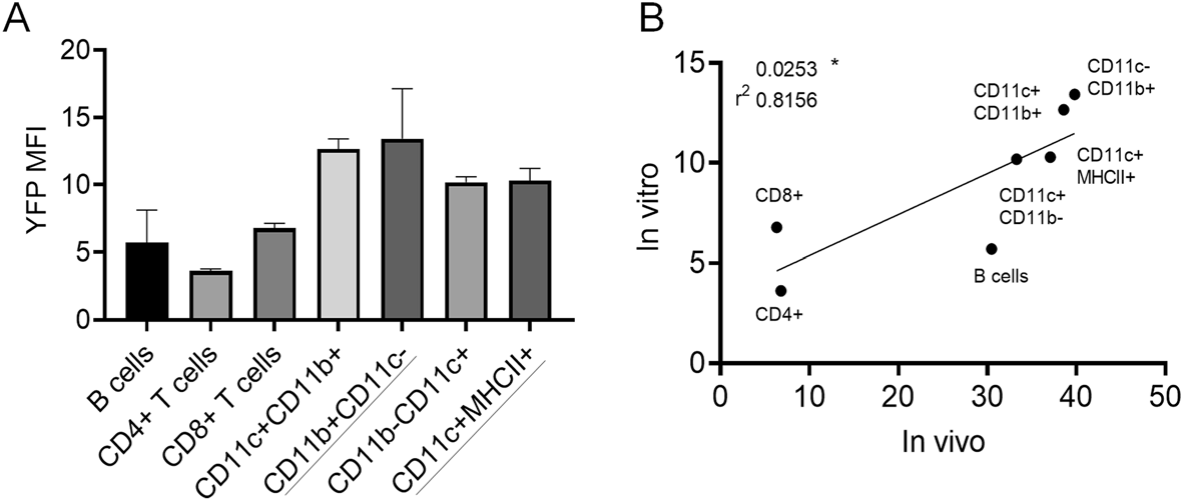
Inducibility of Cre/Esr1 by 4-OHT in different immune cells in vitro correlates with the pattern of induction in vivo. Splenocytes from CAG-Cre/Esr1 mice were cultured with anti-CD3/CD28 and LPS to maximize lymphocyte viability, and with or without 4-OHT (2 μM), for 48–72 h. Because of limited survival of myeloid cells in vitro, we used the maximal YFP induction at either 48 h or 72 h for each population. Some spontaneous induction of YFP (1–2%) was observed in the absence of 4-OHT, possibly an effect from fetal bovine serum components, and this background for each population was subtracted to the %YFP+ induced with 4-OHT. (**A**) %YFP+ cells induced by 4-OHT in gated immune cell populations (all from 72 h time point, except those underlined, from 48 h). Data show the mean ± SEM (from 2 mice). (**B**) Correlation plot for %YFP+ induced in vivo with TAM and in vitro with 4-OHT for different immune cell populations.

In order to more directly assess possible differences in inherent sensitivity to TAM between immune cell populations, we subjected spleen cells to the bioactive metabolite 4-OHT for 48-72 h in vitro^26^, and then assessed YFP induction as we have done ex vivo. We provided non-specific stimulation for T cells and B cells to improve viability. Although viability was more limited, we observed that myeloid cells more efficiently converted to YFP+ cells than lymphocytes (Fig. 7A, Suppl. Information Fig. S5), consistent with observations from in vivo treatments. The efficiency of YFP induction in vivo and in vitro correlated between immune cell populations (Fig. 7B, p = 0.022), although B cells induced YFP to a relatively greater degree in vivo than in vitro.

## Discussion

The TAM inducible Cre/loxP system is widely used for genetic manipulation in vivo to control transgene expression and track individual cells or cell lineages^3,27^. Cre may be expressed either constitutively or conditionally, which is particularly useful when the study requires selective targeting. Cre technology has proven crucial in studies on immune cell tracking, function and development of specific populations of cells. A few examples include generation of CD4-*Cre/ER* transgenic mice to study development of peripheral CD4+ T cells after maturation in the thymus, cytosolic STING pathway in induction of type I IFN by innate cells, and assessing B cell response following selective and inducible over-expression of activation induced cytidine deaminase^26,28,29^.

Optimization of TAM delivery and dosing depends largely on the target site or tissue and concentration of the drug. To date, there is no standardized dose or consistent delivery method of TAM documented for efficient activation of Cre, particularly in cells of the immune system. Here, we describe an optimized induction method of Cre with TAM by measuring the induction rate of Cre using the YFP reporter and quantifying TAM concentration in tissues and serum. Utilizing Cre/ER mice with inducible YFP reporter, we documented that TAM activation of Cre is dose-dependent and greater when given IP. This observed effect of TAM persisted even after discontinuing administration, leading to increased Cre induction 3–4 weeks following the start of TAM dosing, which may be due to a gradual release of TAM from the corn oil and/or the stability of TAM in vivo.

In addition to the desired phenotypic changes from Cre-induced recombination, administration of TAM may have side effects that complicate interpretation of the biological results of gene ablation or overexpression. This does not include induction of YFP, which is not expected to be subject to confounding results. In the current study, high dose IP delivery of TAM caused long-term toxicity, resulting in morbidity and a high rate of mortality in mice. However, there may be confounding effects of TAM on mice due to the delivery vehicle, which should be addressed in future studies. IP administration of pharmaceutical-grade corn oil caused peritonitis in mice given four total injections every other day^30^. Although no reports have indicated any toxic effects of corn oil-formulated TAM, we can at least in part ascribe the observed hepatotoxic effect to the oil, which leads to hepatic lipidosis in rats^31^ and fatty livers in mice supplemented with corn oil over 42 days^32^. There have also been previously described changes in hepatic metabolism due to TAM administration in oil^33^. The inherent toxicity of different types of oil should be comparatively assessed in future studies to minimize these adverse effects. While TAM-supplemented diets can circumvent this issue, dosing is more limited and cannot be controlled, and other adverse effects, such as weight loss, are observed from poor feeding due to aversion. Additionally, toxic effects of TAM administered at high doses (90 µg/g IP) have been previously described, reporting adverse effects of Cre toxicity^34^. After noting these effects, the group titrated the TAM dose down to 30 µg/g for three consecutive days to minimize mortality and maximize recombination, resulting in optimal induction of Cre recombination of the desired gene in cardiomyocytes^34^.

In this study, treatment with 6 mg TAM IP induced liver injury, leukocytosis, and increased liver enzymes. The optimal dose for efficient induction of Cre by day 21 was 3 mg PO once daily for 5 consecutive days. In a study in which compounds were injected IP every 48 h for a total of 4 injections, WBC were all within normal limits and there were no changes in body condition score or visual assessment score. Additionally, the study did not report vacuolation in organs assessed via histopathology^30^. So far, IP has been the delivery method of choice for TAM in the majority of studies because the amount administered can be better controlled^35^. However, delivery via oral gavage is also possible, but the efficacy of Cre induction and in vivo affects using this method have not been previously fully explored. Here we document that a lower dose of TAM (3 mg) is safe when delivered PO, with no mortality and minimal impact on the liver while still inducing efficient Cre activity. According to our observations, these side effects appeared reversible upon efficient elimination of TAM in serum and tissues and resulted in normalization of tissue pathology up to 4 weeks after initiation of treatment.

Even under the best conditions, based on our optimized TAM administration, induction of Cre (measured by YFP) was uneven between different immune populations. Myeloid cells had the highest level of Cre/YFP induction (30–40%), followed by B cells (30%) and T cells (7–14%). For unclear reasons, induction of Cre/YFP was nearly double in thymocytes compared to peripheral T cells, and was particularly high in double positive (DP) cells (nearly 25%). One way to study any tissue disparity in the activation of Cre is to measure TAM levels in tissues of interest. We first analyzed TAM accumulation and clearance in peripheral blood serum samples. Maximal accumulation of TAM occurred one week after initiating treatment, followed by a slow decrease in concentration that was complete by 28 days, regardless of dosing or delivery method. Interestingly, there was a parallel increase in expression of the YFP reporter, revealing that Cre activity increases most dramatically within the first week after initiating treatment, and then steadily rises at a slower rate despite discontinuation of TAM dosing. Next, we measured the concentration of TAM in various tissues (liver, spleen, LNs, thymus) and found no preferential accumulation of TAM in specific tissues that could explain the unequal Cre induction rates in distinct immune populations. The disparity of TAM accumulation in different tissues may suggest that immune cells can process TAM differently. Myeloid cells induced Cre/YFP at higher rate than lymphoid cells, particularly in the spleen and peripheral blood, which harbor a greater proportion of myeloid cells than LNs and thymus. The higher Cre recombinase activity in myeloid cells may be the reflection of an elevated metabolic activity relative to lymphocytes, which are mostly naïve and quiescent.

The disparity of Cre expression between cell types may be the result of one or several combined factors. One such factor is the activity of the CAG promoter driving Cre expression. Functional analysis of various promoters at different stages of embryonic stem cell development has been previously documented^36^. Inducible expression of genes was shown to be variable across and between different stages of hematopoietic lineages. Specifically, Tat-regulated expression of the CAG promoter resulted in efficient reporter expression throughout T cell development in the thymus, especially in immature and developing T cells^37^. These findings may partly explain the increased induction of Cre in DP thymocytes. The higher rate of YFP induction in DP thymocytes relative to other T cell subsets is particularly interesting, and further study is needed to assess if this is facilitated by active T cell receptor recombination. Another factor to consider is the expression level of endogenous ER (Esr1 and Esr2), which could compete with the transgenic Cre/ER to bind 4-OHT. However, this possibility is unlikely given that the mutation in the transgenic ER provides a strong affinity for 4-OHT and therefore a binding advantage. Moreover, the expression profile of Esr1 in different immune cells does not inversely correlate with Cre/YFP induction as would be expected if competition for receptor sites occurred. Esr2, on the other hand, is not detected in immune cells or liver, rather it is primarily expressed in the ovaries and prostate of mice. A third explanation of altered expression in DP thymocytes is a differential ability of cells and tissues to take up and metabolize TAM. As previously discussed, TAM is converted to 4-OHT mainly in the liver by the products of the highly expressed Cyp2d9 and Cyp2d10 genes^38^. Expression of these genes in immune cells is negligible and therefore cannot explain why certain immune cells respond differently to TAM. Our in vitro data showed that, in conditions where 4-OHT is non-limiting, there remains an inherent limitation for immune cells, particularly T cells, to induce Cre-mediated recombination. Although this remains to be established, it could reflect different levels of CAG promoter activity driving Cre/ER between these cells. In this model, Cre induction is indirectly assessed based on YFP induction, and once Cre activity removes the Stop codon before YFP, its expression is controlled by the endogenous Rosa26 promoter. Different reporters show variable sensitivity to Cre^39^, and it is conceivable that this sensitivity varies between cell types due to unequal Rosa26 locus activity and accessibility to Cre recombinase. It is important to keep in mind that Cre activity in one site (reporter induction) may not guarantee that recombination at another site (e.g. target gene to be ablated) always occurs, as disparities exist at this level as well.

Aside from the disparity between tissues, one may wonder whether the overall lower Cre inducibility, in comparison to other studies, reflects a relative resistance of cells of the hematopoietic lineage or a different type of ER used. Concerning ER type, many studies have utilized Cre/ERT2, which has a modified human ER ligand binding domain, whereas the Cre/Esr1 used here has a modified mouse ER ligand binding domain. To our knowledge, a direct comparison on the efficiency of ERT2 and Esr1 in vivo has not been done, but it is possible that Esr1 has a lower sensitivity to 4-OHT. ERT2 is an improved version of ERT, whereby two additional mutations conferred a tenfold increase in sensitivity to 4-OHT^1^. Disparities between tissues exist in Cre induction for both Cre/Esr1 and Cre/ERT2 with ubiquitous promoters (e.g. Jax strains B6.Cg-Tg(CAG-cre/Esr1)5Amc/J (004682) and B6.Cg-Tg(UBC-cre/ERT2)1Ejb/J (008085) as reported on The Jackson Laboratory website); however our study is the first to report disparities among immune cells in a ubiquitous inducible model.

The observation that both the percentage and levels of YFP continue to increase long after discontinuing TAM treatment suggests that TAM may somehow be stored and released over time. Our pathology data demonstrate a substantial accumulation of lipid vacuolation in the liver and spleen, which resolves over time. These lipid droplets may serve to store TAM and slowly release it as they are broken down, primarily by myeloid cells such as macrophages. This can give myeloid cells greater accessibility to TAM/4-OHT. Furthermore, these cells have greater endocytic capacity and are more rapidly replenished by stem/progenitor cells, thus it would be interesting to assess Cre/ER activity in hematopoietic stem/progenitor cells as well.

We have determined optimal conditions (dose and delivery route of TAM) for efficient induction of Cre activity in immune cells, based on expression of the YFP reporter. These optimal conditions constitute a compromise between increased induction of YFP in immune cells and minimal toxicity in vivo. Nonetheless, these conditions may still have biological effects that are independent of those resulting from ablation of the target gene. Thus, proper controls (oil only, TAM with oil, including mice without Cre or with non-floxed alleles) remain critical in gene ablation studies. This work highlights underappreciated disparities in the induction of Cre activity in different immune cells using a ubiquitous TAM-inducible model, demonstrating that gene ablation can be most efficiently achieved in myeloid cells and is more limited in T lymphocytes, despite similar TAM accumulation in different lymphoid tissues.

## Methods

### Mice

C57BL/6 (B6) mice were purchased from Charles River Laboratories. Cre/ER-transgenic mice (B6. Cg-Tg(CAG-cre/Esr1*)5Amc/J, #004682) and Cre-induced YFP reporter mice (B6. 129X1-Gt(ROSA)26Sor <tm1(EFYP)Cos> /J, #006148) on a B6 background were from The Jackson Laboratories. Cre/ER transgenic mice were crossed with YFP transgenic mice. Mice were acclimated for at least 3 days prior to experimental use. Animals were housed at a maximum of 5 per cage in individually ventilated microisolation cages with autoclaved corncob bedding. Both male and female mice were used at 8–20 weeks of age in all experiments. Mice were assigned to cages according to treatment. Enrichment was provided in the form of social housing and cotton nesting pads. Mice were maintained in a temperature-controlled environment (20–26 °C), fed irradiated chow (Purina Lab Diet 5053, PMI, St Louis, MO) and given unrestricted access to acidified water. Mice were maintained in a SPF facility in accordance with the Guide for the Care and Use of Laboratory Animals in an AAALAC-accredited facility.

### TAM preparation and treatment

For the first experiment, 1.2 mg and 2.4 mg TAM was prepared from powder (Sigma, T5648) dissolved in 100% ethanol (biology grade) (ThermoFisher Scientific BP2818100) at 100 mg/mL. Then, 1 mL of ethanol with TAM was mixed with 7 mL of corn oil (Acro Organics 405,435,000) for a final concentration of 12.5 mg/mL. Mice were treated with 5 injections every other day (over 10 days). With this protocol, 2.4 mg was the highest dose we could inject per mouse. For subsequent experiments with 3 mg and 6 mg doses, TAM was purchased from ApexBio (B5965). TAM was dissolved directly in corn oil at 50 °C and filtered through sterile syringe filter (0.2 μm PES, Millipore Sigma). This method allowed more concentrated TAM–oil mixtures to be produced. Briefly, TAM was prepared in 10 mL filtered corn oil. 300 mg TAM was added to the oil and the solution was placed on a shaker for 2 h at 50 °C until completely dissolved for a final concentration of 30 mg/mL. Mice were treated for a period of 5 days either IP or by oral gavage (PO) with 0.1 mL (3 mg) or 0.2 mL (6 mg).

### TAM serum and tissue concentration measurement

The assay to measure TAM serum and tissue concentrations was performed by Biomarkers Core Laboratory (BCL) at the Irving Institute for Clinical and Translational Research. Measurement of TAM concentration from serum and tissues was achieved using Ultra Performance Liquid Chromatography-Tandem Mass Spectrometry (UPLC-MSMS). The internal standard was purchased from Sigma-Aldrich (St. Louis, MO) and the deuterated internal standard was procured from TRC Chemicals (Ontario, Canada). Liquid–liquid extraction was utilized to isolate TAM by mixing 50 µL of either mouse serum or tissue homogenate combined with the internal standard (TAM Day 5) with 3 mL of a 3-to-2 hexane/dichloromethane mixture. The mixture was vortexed for 10 min at 2000 rpm and then centrifuged for 10 min at 2,200 rpm. The aqueous phase was frozen in liquid nitrogen and then the organic layer was decanted and evaporated. The remaining extract was suspended in 1 mL of dichloromethane, transferred to a Liquid chromatography–mass spectrometry (LCMS) vial, evaporated under a nitrogen stream and resuspended in 50% methanol for LCMS analysis.

LCMS analysis was performed on a Waters Xevo TQS MS integrated with a ACQUITY UPLC system (Waters, Milford, MA, USA) controlled by MassLynx Software 4.1. The sample (5 µL) was loaded onto a 50 °C Waters Acquity UPLC BEH C18 (1.7 um 2.1 × 100 mm) with a flow rate of 500 μL/min. The initial conditions set were 75% phase A (water with 0.1% formic acid) and 25% mobile phase B (acetonitrile containing 0.1% formic acid). Solvent B was increased linearly to 95% over 7 min and maintained through 7.5 min with a total run time of 8 min. Positive ESI–MS/MS with multiple reaction monitoring (MRM) mode was performed. The following MRM transitions were used: TAM 372.2 > 129.0 and TAM D5 376.7 > 133.6. TAM concentration was quantified by comparing integrated peak areas against established amounts of purified standards. The method’s lower limit of quantitation was 0.1 ng/mL. The intra-assay and inter-assay precision was 2.21% and 4.20% respectively.

### Histopathology, hematology and clinical chemistry

In order to assess morphological changes of tissues after TAM administration livers, LNs and spleens were fixed in 10% formalin and submitted to Bolder Biopath (Colorado, USA) for analysis. Formalin-fixed sections of the above tissues were embedded in paraffin, sectioned at 5 microns and stained with hematoxylin and eosin. Slides were evaluated for evidence of lymphoid depletion, degeneration, necrosis, and any other lesions that would be consistent with an adverse reaction to TAM or the delivery vehicle (corn oil). Other changes considered incidental or not related to the administration of TAM were also described.

To analyze hematology and clinical chemistry, whole blood was collected at the time of euthanasia via cardia puncture and pooled based on TAM treatment into tubes containing EDTA and clot activator and serum gel separator. In some cases, when the amount of blood was insufficient for those assays, blood from two or more mice from the same group was pooled. The Complete Blood Count was analyzed using a Genesis Hematology System (Oxford Science). The serum chemistry analysis was conducted using a calibrated Heska Element DC Chemistry Analyzer.

### Analysis of immune cell populations in different lymphoid tissues

To analyze TAM concentration and Cre activity in peripheral blood of animals, mice were bled from the tail vein and blood was collected in tubes containing heparin (1,000 USP Units/mL; McKesson NDC 63739-931-14). Red blood cells were lysed with 1 mL 1 × ammonium-chloride-potassium (ACK) lysis buffer (Gibco, A10492-01) and the remaining cells were resuspended in 100 μL FACS buffer, containing 1 × PBS, 20% fetal bovine serum (FBS) and 0.05% sodium azide (Sigma-Aldrich S2002). To measure TAM accumulation and Cre induction in immune cell populations from primary and secondary lymphoid organs, mice were euthanized and dissected to collect LNs, spleen, thymus and bone marrow. LNs and splenocytes were processed by triturating tissues between frosted glass slides and collected in filtered complete RPMI 1640 medium (Corning 15-040-CV) supplemented with 10% fetal bovine serum, 5,000 I-U/mL penicillin, 5,000 u/mL streptomycin (Corning), sodium pyruvate (100 nM, Corning 11360-70), non-essential amino acids (0.1 mM, Corning 11140-050), l-glutamine (200 mM, Corning 25-005-CI) and β-mercaptoethanol (50 μM). Tissue samples were lysed with 2 mL ACK buffer and filtered through a 70 µm cell strainer. Samples were washed with complete medium and resuspended 5 mL of medium for counting. 1 × 10^6^ million cells were stained in 100 μL volume of FACS buffer with the following anti-mouse monoclonal antibodies: CD45 (APC-Cy7, clone 30-F11), CD3 (PE-Cy7, clone 145-2C11), CD45R/B220 (AF700, clone RA3-6B2), CD4 (PE, clone GK1.5), CD8 (BV510, clone 53-6.7), CD11c (PE, clone N418), CD11b (Pacific Blue, clone M1/70), MHC-II (AF700, clone M5/114.15.2), biotinylated Gr-1 (Ly-6G/Ly-6C) (clone RB6-8C5, detected by streptavidin (APC)), anti-mouse CD16/32 FcR block (clone93), propidium iodide. All antibodies and dyes were from Biolegend. Cells were incubated for 30 min with antibodies at room temperature, washed once with FACS buffer and kept on ice until acquisition. Samples were acquired on FACSCantoII and Fortessa from Becton Dickinson (BD), followed by data analysis using FlowJo software (version 10; BD). Frequencies of YFP+ cells from various lymphoid tissues (LNs, spleen, peripheral blood and bone marrow) were quantified on gated T/B lymphocytes (Suppl. Information Fig. S2A–E) and myeloid cell populations (Suppl. Information Fig. S2F,G), and from thymic tissue on gated double positive (DP), double negative (DN) and single positive (CD4+/CD8+) thymocytes (Suppl. Information Fig. S2H).

### In vitro treatment with 4-OHT

Culture plates (flat bottom 96-well) were pre-coated with anti-CD3 (clone 145.2C11) and anti-CD28 (clone 37.51), each at 1 µg/mL for 2 h at 37 °C, and then washed twice. Splenocytes from CAG-Cre/Esr1 mice were subjected to density gradient isolation (Lymphoprep; StemCell Technologies, # 07801; per manufacturer’s instructions) and treatment with ACK buffer. Isolated cells were plated at a density of 4 × 10^5^ cells per well (6 replicates per mouse) in complete RPMI medium. The culture medium was supplemented with 100 ng/mL LPS (*Escherichia coli* 026:B6; eBioscience #00-4976-03) with or without 2 µM 4-OHT (Cayman Chemical, #14853). After 48 h, 3 replicates were collected and medium of the other 3 replicates was refreshed (including LPS ± 4-OHT) for an additional 24 h of culture. For each time point, flow cytometry was performed using antibodies to CD3, CD4, CD8, B220, CD11b, CD11c, MHC-II, as well as FcR block and propidium iodide, from the above list. Samples were acquired on BD Fortessa and data was analyzed using FlowJo 10.6.2 (BD).

### Statistical analysis

To identify significant differences in induction of YFP+ cell frequencies (within CD45+ cells), TAM serum concentrations and fraction of weight loss between different groups of TAM-treated mice (1.2 mg vs 2.4 mg; 3 mg vs 6 mg) either by IP or PO, we applied mixed model ANOVA tests with repetitive measures (Figs. 1B, 2A–C, 3A, 4A,B). To compare percentages of YFP+ among CD45+ cells in 3 mg TAM PO treated mice between day 5 and other days of measurement, we used non-paired T cells for unpaired samples (Fig. 4C,D). Non-paired samples as presented in Fig. 3C,D were compared using Mann–Whitney test. Survival rate between different groups of mice treated with 3 mg vs 6 mg either IP or PO were compared by Log rank test (Fig. 2D). TAM concentration in different tissues from different experimental groups: 3 mg vs 6 mg treated mice with IP or PO were tested by Mann–Whitney test (Fig. 5A–F). Same test (Mann–Whitney) was applied to identify differences in YFP induction among lymphoid lineage cells (Fig. 6A–E) and myeloid cells (Fig. 6F–K) across different tissues. Statistical analyses were performed on Prism 8, version 8.1.0. *p ≤ 0.05, **p ≤ 0.01, ***p ≤ 0.001, ****p ≤ 0.0001.

### Ethics statement

Animal experiments were carried out at the Columbia Center for Translation Immunology, under a protocol reviewed and approved by Columbia’s Institutional Animal Care and Use Committee (IACUC).

## Supplementary information

Supplementary Information

## Data Availability

The datasets generated during and/or analysed during the current study are available from the corresponding author on reasonable request.
