# Supplementary material for: Optimization of tamoxifen-induced Cre activity and its effect on immune cell populations
Source: Sci Rep. 2020 Sep 17;10:15244. doi: 10.1038/s41598-020-72179-0 (PMC7499195; doi:10.1038/s41598-020-72179-0)
Supplement: Supplementary file 1 — Supplementary Information [file 41598_2020_72179_MOESM1_ESM.pdf]

Optimization of tamoxifen-induced Cre activity and its effect on immune cell populations

Rachel S. Donocoff, Nato Teteloshvili, Hyunsoo Chung, Rivka Shoulson, Remi J. Creusot

Suppl. Information Fig. 1

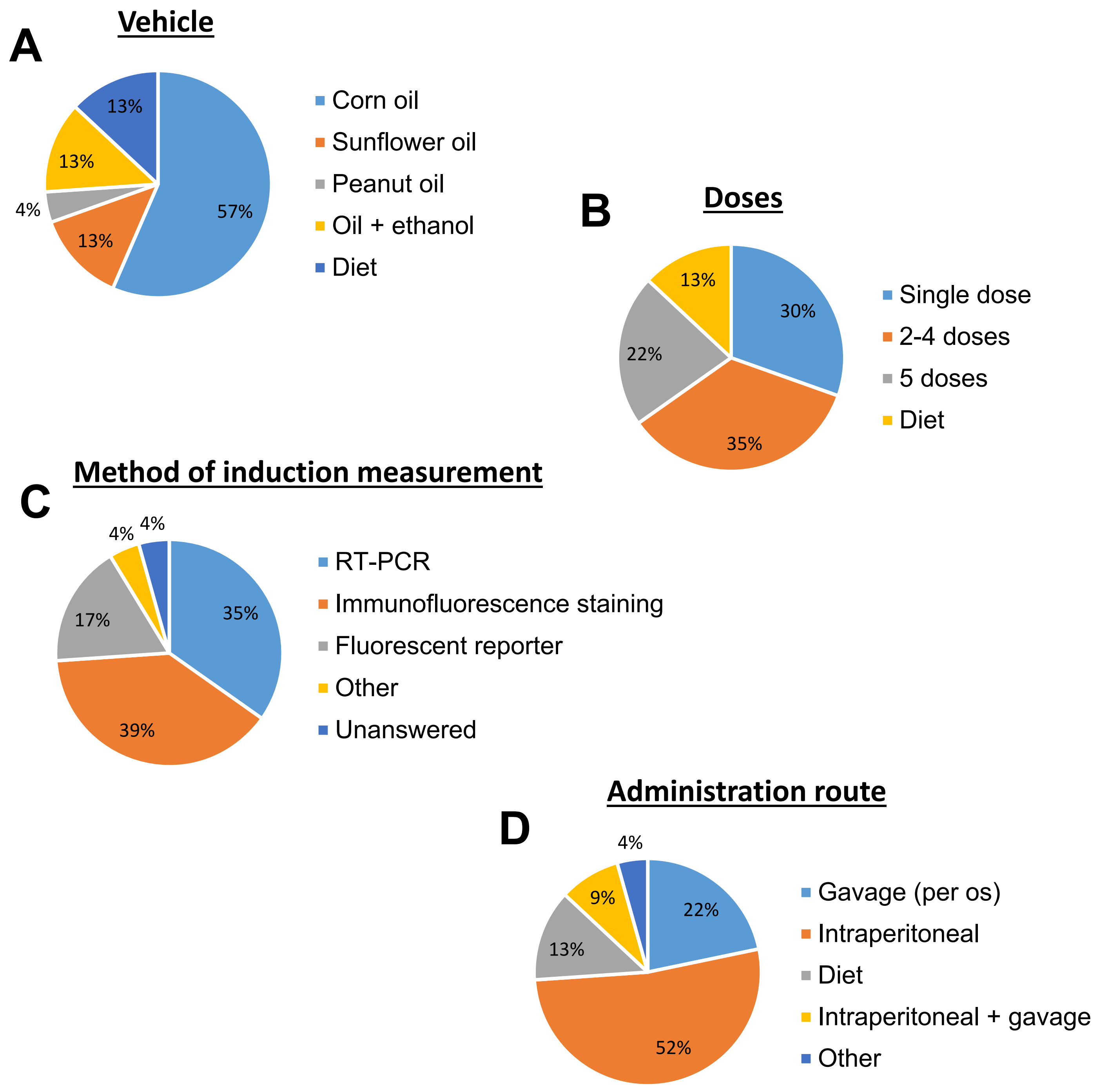

Suppl. Information Figure 1. Results of an internal survey of Columbia University researchers using Tamoxifen in their animal protocol. Researchers who listed Tamoxifen as a drug in their IACUC animal use protocol were surveyed with the following questions: 1) How does your lab formulate tamoxifen and what vehicle do you use? 2) How is Tamoxifen administered to your mice? 3) What is the number of doses typically administered to animals? 4) How are you measuring the induction rate of the inducible gene of interest for your study? There were 23 respondents who were actively using Tamoxifen in mice. Responses are shown for vehicle (**A**), number of doses (**B**), method of induction measurement (**C**) and administration route (**D**).

Suppl. Information Fig. 2

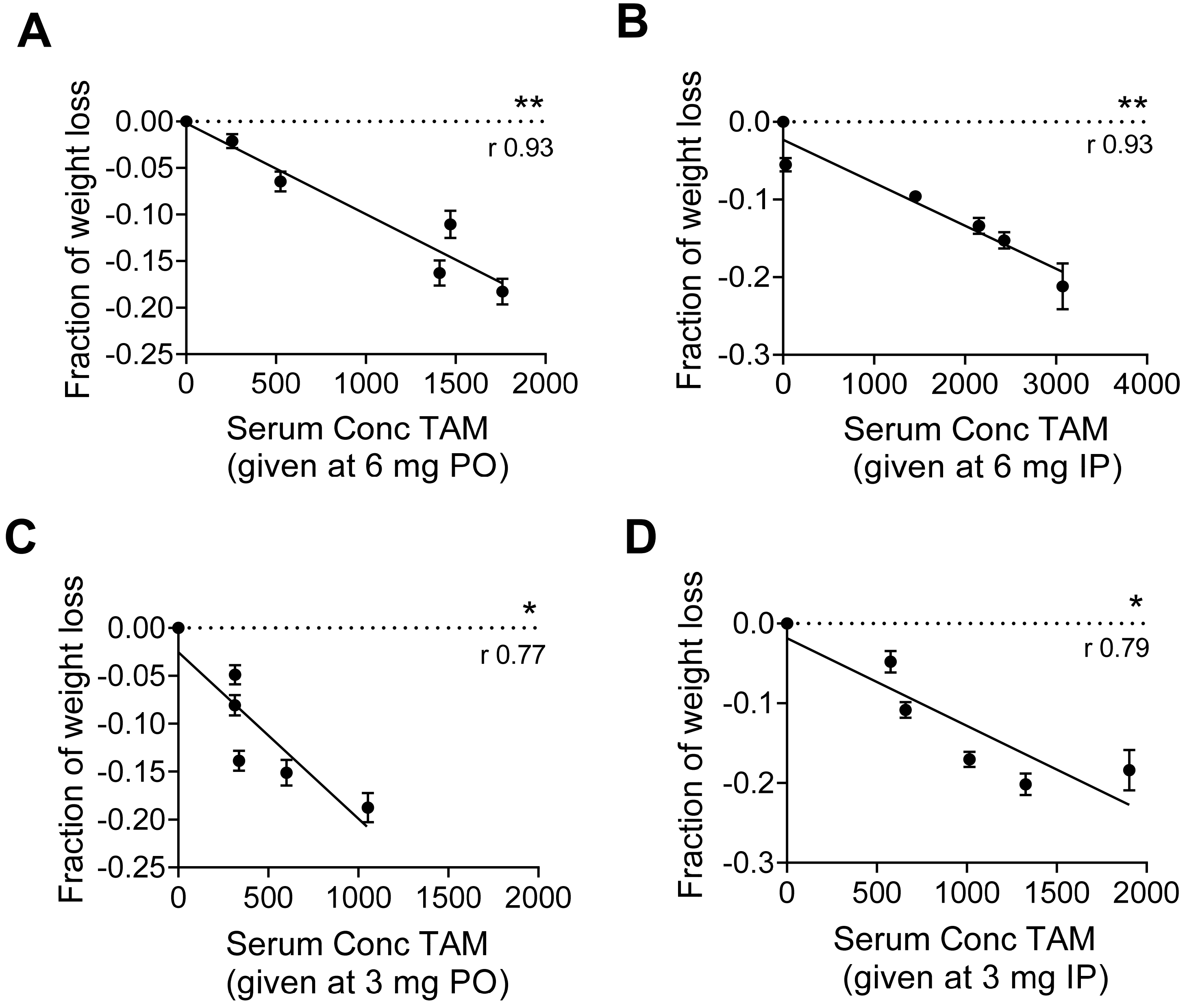

Suppl. Information Figure 2. Correlation between fraction of weight loss and concentration of TAM found in serum of animals treated with either 6 mg PO (**A**), 6 mg IP (**B**), 3 mg PO (**C**) or 3 mg IP (**D**).

Suppl. Information Fig. 3

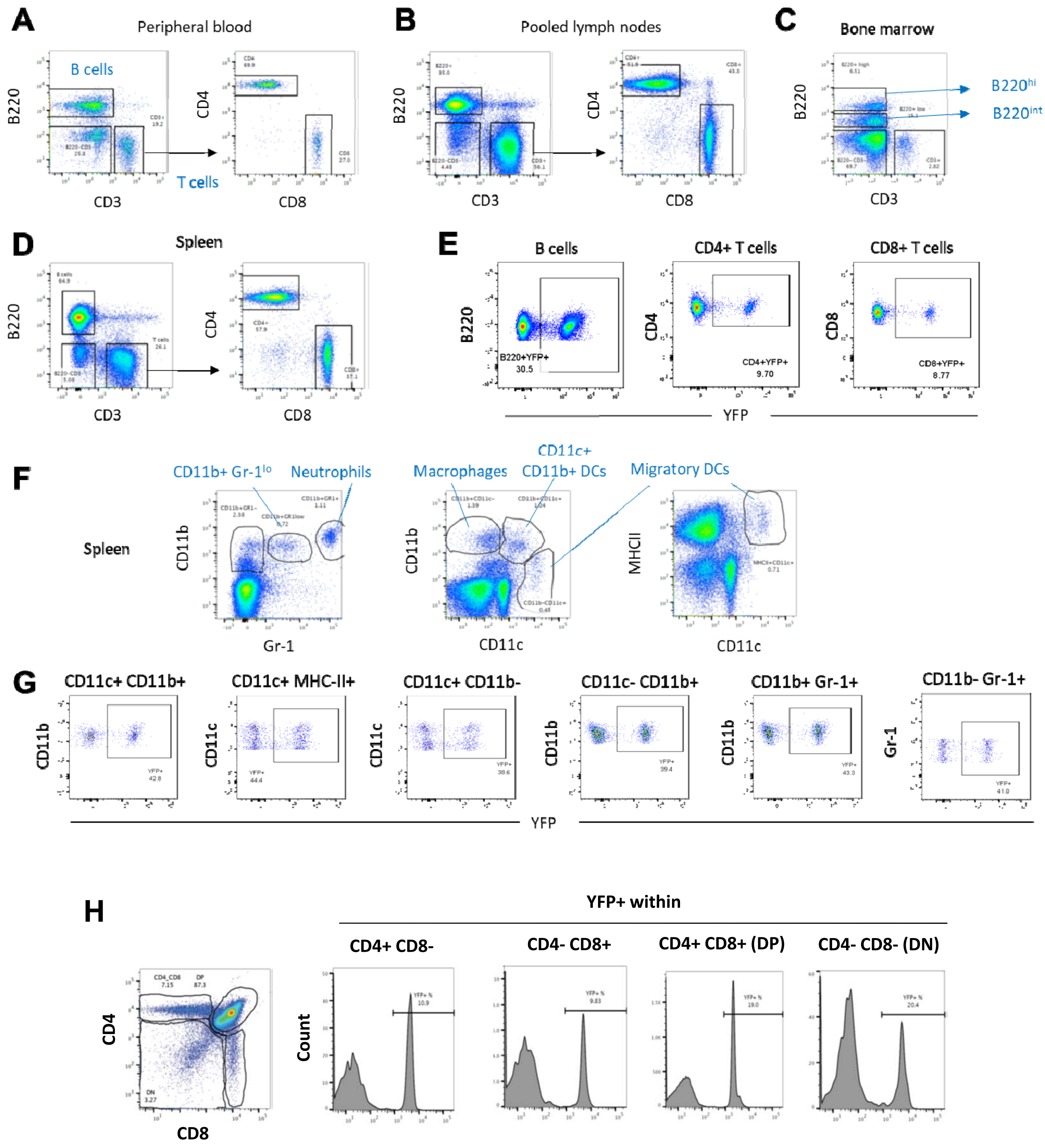

Suppl. Information Figure 3. Phenotypic analysis of cells inducing YFP expression by TAM-mediated recombination of Cre in vivo. Gating strategy to analyze YFP induction in B cell / T cell populations in peripheral blood (**A**), pooled lymph nodes (**B**), bone marrow (**C**), spleen (**D**), with representative YFP expression (**E**), and in various myeloid cell populations in spleen (**F**), with representative YFP expression (**G**). Gating strategy to identify YFP+ cells among thymocytes, single positive CD4+ and CD8+, double positive (DP) and double negative (DN) cells (**H**).

Suppl. Information Fig. 4

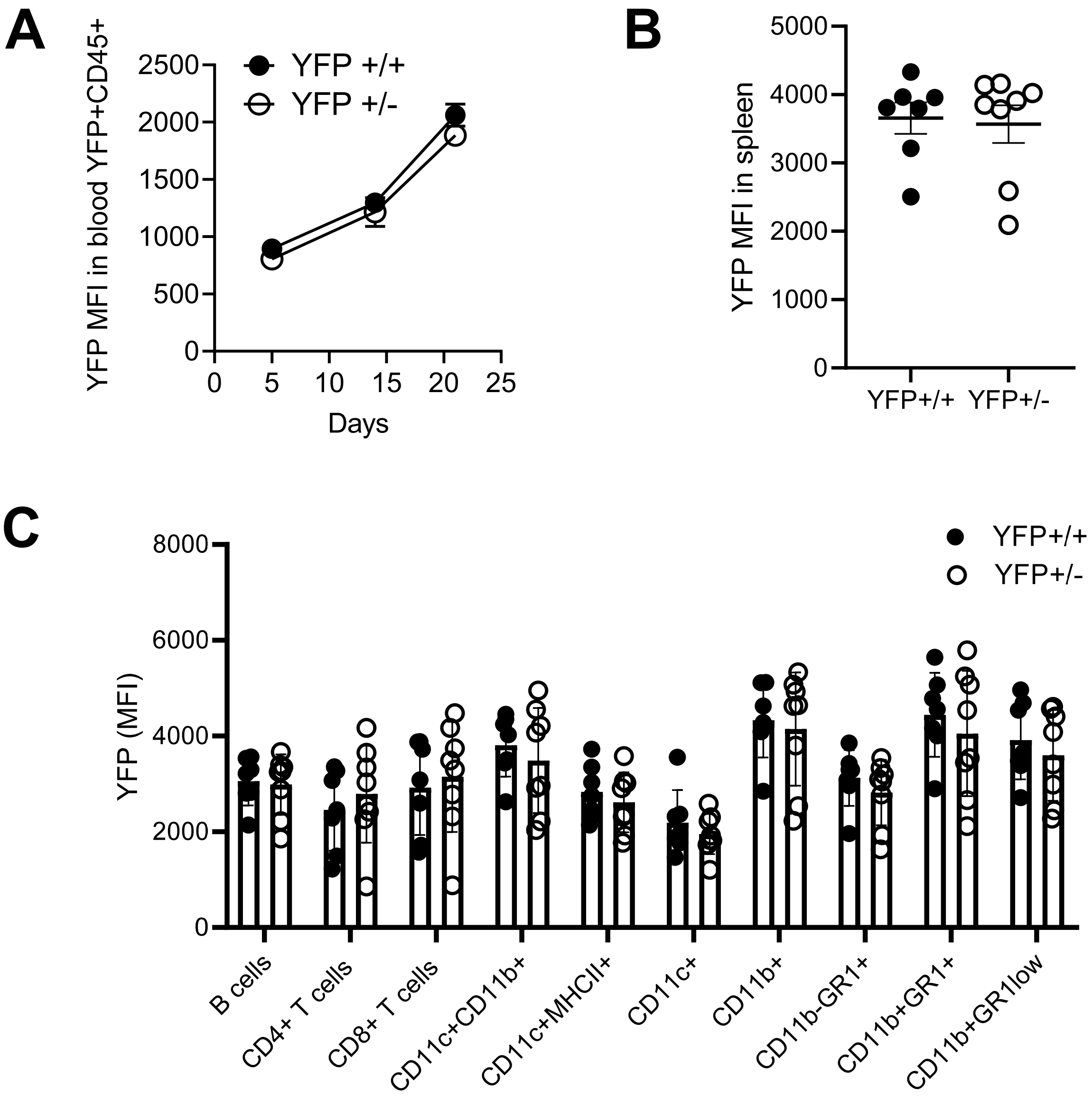

Suppl. Information Figure 4. Variation of YFP expression levels between cell types and based on number of copies of YFP reporter transgene. Mice were analyzed separately based on their genotype at the level of the YFP reporter transgene (+/+ homozygous; +/- heterozygous). YFP mean fluorescence intensity (MFI) was analyzed on YFP+ cells in peripheral blood CD45+ cells during monitoring (**A**) and in splenocytes (gated on live singlets) at endpoint (32 days after beginning of treatment) (**B**). Data show the mean ±SEM from 6-8 mice per group. For the data shown, all mice were analyzed on the same day , enabling MFI comparison. The level of YFP in different subsets of immune cells was assessed in the spleens from YFP+/+ and YFP +/- mice after 32 days, over 3 different days (**C**).

Suppl. Information Fig. 5

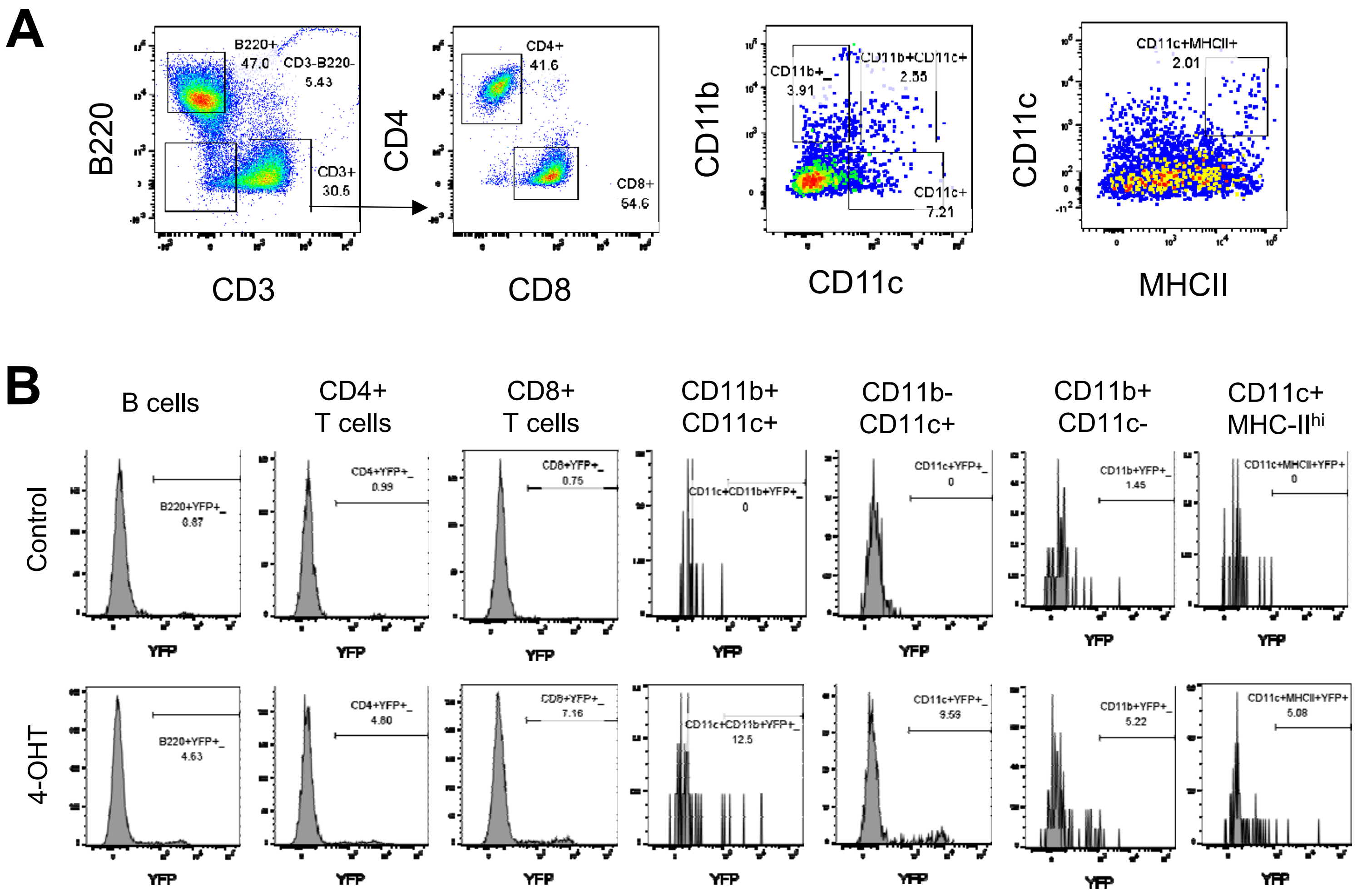

Suppl. Information Figure 5. Phenotypic analysis of cells inducing YFP expression by 4-OHT-mediated recombination of Cre in vitro. (**A**) Gating strategy to analyze YFP induction in B cell / T cell and myeloid cell populations in splenocytes collected after 72h of culture on anti-CD3/CD28 and with LPS with or without 4-OHT. YFP expression in samples with or without 4-OHT (2 μM) after 72h of treatment (48h for the last two populations) (**B**).

Suppl. Information Table 1

A

Cell population	Esr1	Esr2	Cyp2d9	Cyp2d10	Cyp2d11
B cell (spleen)	29.15	2.12	0.10	0.78	0.1
CD4 T (thymus)	7.69	0.10	0.10	0.55	0.32
CD8 T (thymus)	18.61	0.63	0.10	0.1	0.59
DP (thymus)	36.85	1.33	0.10	1.33	0.51
CD4 T naïve (spleen)	43.10	0.73	0.10	1.05	0.1
CD8 T naïve (spleen)	40.61	0.10	0.10	0.1	0.1
CD8+ DC (spleen)	5.58	4.40	0.10	2.32	1.14
CD4+ DC (spleen)	44.81	2.57	1.34	0.58	0.34
Granulocytes (spleen)	31.49	1.95	0.1	0.1	0.1
Macrophage (spleen)	87.80	2.42	0.1	0.1	0.1

Immgen database. ULI RNASeq

B

Cell population / tissues	Esr1	Esr2	Cyp2d9	Cyp2d10
B cell (follicular)	16.38	4.64	4.79	4.99
CD4 T (thymus)	15.54	4.64	7.85	4.64
CD8 T (thymus)	16.83	4.64	8.99	7.47
DP (thymus)	16.31	4.64	5.29	4.64
CD4 T	15.54	4.64	4.64	4.64
CD8 T	19.48	4.64	8.06	5.37
CD8a+ DC	6.65	4.64	6.47	4.64
CD8a- DC	12.06	4.64	6.52	5.06
Granulocytes	15.54	4.64	4.64	4.64
Macrophage (bone marrow)	15.61	4.64	5.32	8.18
Spleen	11.92	4.64	4.64	4.64
Lymph nodes	18.21	4.64	4.64	4.64
Bone marrow	29.71	4.64	4.64	4.64
Liver	244.59	4.84	23,070.48	16,041.52

BioGPS database

Suppl. Information Table 1. Esr1, Esr2 and Cytochrome P450 (2d9-11) gene expression by deep RNA-seq (Gene Skyline, ImmGen ULI RNASeq, http://www.immgen.org/) (**A**) and microarray (BioGPS database, http://biogps.org/) (**B**). Probe sets used for BioGPS data were 1435663_at (Esr1), 1426103_a_at (Esr2), 1419349_a_at (Cyp2d9) and 1418113_at (Cyp2d10). No data available on BioGPS for mouse Cyp2d11.
